# The protective effect of Apelin-13 against cardiac hypertrophy through activating the PI3K-AKT-mTOR signaling pathway

**DOI:** 10.22038/IJBMS.2022.65160.14356

**Published:** 2023-02

**Authors:** Yu Peng, Ruan Jingming, Chen Shaowen, Huang Feng, Zhu Pengli

**Affiliations:** 1 Provincial Clinical Medical College of Fujian Medical University, Fuzhou 350001, Fujian, China; 2 Department of Cardiovascular Medicine, Fujian Provincial Hospital South Branch (Fujian Provincial Jinshan Hospital), Fuzhou 350028, Fujian, China; 3 Department of Geriatric Medicine, Fujian Provincial Hospital, Fuzhou 350001, Fujian, China; 4 Fujian Provincial Institute of Clinical Geriatrics, Fujian Key Laboratory of Geriatrics, Fujian Provincial Center for Geriatrics, Fuzhou, 350001, Fujian, China; # These authors contributed eqully to this work

**Keywords:** AKT, Apelin-13, Cardiac hypertrophy, mTOR, PI3K

## Abstract

**Objective(s):**

To determine the protective effect of Apelin-13 on cardiac hypertrophy through activating the PI3K-AKT-mTOR signaling pathway.

**Materials and Methods:**

The phenylephrine-induced cardiomyocyte hypertrophy model was established in H9C2 cells *in vitro*. Electroporation transfection technology was utilized to prepare and screen the H9C2 cells inducing low expression of the angiotensin type one receptor-related protein (Si-APJ). H9C2 and Si-APJ cells were divided independently into five groups: the control group, the PE group, the PE+Apelin group, the PE+Rapa group, and the PE+Apelin+Rapa group. RT-PCR was performed to analyze the mRNA expression levels of myosin heavy chain 7 (MYH7). Expression of the PI3K/AKT/mTOR pathway proteins and MYH7 was investigated by western blot.

**Results:**

The expression of PI3K/AKT/mTOR phosphorylated proteins was significantly higher in the PE group compared with the PE+Apelin group in H9C2 cells (*P<*0.05). Conversely, in Si-APJ H9C2 cells, the expression of PI3K/AKT/mTOR phosphorylated proteins was decreased (*P<*0.05). In H9C2 cells, the expression of MYH7 protein was increased in the PE group compared with the control group (*P<*0.05). In the same cell line, the expression of MYH7 in the PE+Apelin group was decreased significantly compared with the PE group (*P<*0.05). In Si-APJ H9C2 cells, compared with the control group, the expression of MYH7 in the PE group still increased significantly (*P<*0.05). In contrast, in the same cell line, there was no statistically significant difference in MYH7 expression between the PE+Apelin, PE+Rapa, and PE+Apelin+Rapa groups compared to the PE group (*P>*0.05).

**Conclusion:**

Apelin-13 reduces PE-induced cardiac hypertrophy by activating the PI3K/AKT/mTOR signaling pathway.

## Introduction

Apelin is a type of polypeptide molecule required for normal blood vessel growth and is synthesized and secreted by vascular endothelial cells. It represents the endogenous ligand of the angiotensin I receptor protein related to the angiotensin type 1 receptor (APJ). Together Apelin and APJ constitute the Apelin/APJ system, which is distributed in the cardiovascular, central nervous, respiratory, and genitourinary systems. It also regulates several physiological processes, such as the cardiovascular system, energy metabolism, fluid homeostasis, neuroendocrine stress response, inflammation, and more (1). Apelin-13 has been identified as a negative regulator of myocardial hypertrophy. Therefore, stimulating the secretion of Apelin-13 could be an important therapeutic approach to inhibit myocardial hypertrophy (2). However, the mechanistic mode of action of Apelin-13 on cardiomyocytes is not yet fully understood.

Cardiomyocyte hypertrophy is the compensatory response of cardiomyocytes to increased cardiac volume and pressure load (3). However, persistent cardiomyocyte hypertrophy is closely related to heart failure, malignant arrhythmia, and sudden cardiac death (4). Seemingly, precluding persistent cardiomyocyte hypertrophy is essential for preventing and controlling hypertrophic cardiac diseases (5, 6). 

To date, several signaling pathways involved in cardiomyocyte hypertrophy, such as PI3K/AKT/mTOR (7, 8), IGF1-PI3K-AKT (9, 10), ERK/p38/NFκB (11, 12), miR-302e/DKK1/Wnt/ beta-catenin (13), and Calcineurin/NFAT (14) have been identified. Among these, the phosphoinositide 3-kinase (PI3K)-Akt-mammalian target of rapamycin (mTOR) signaling pathway (PI3K-Akt-mTOR signaling pathway) is the most common pathway (7, 15, 16), which consists mainly of three action molecules, PI3K, AKT, and mTOR. Physiologically, the PI3K-AKT-mTOR signaling pathway plays a key role in cell proliferation and apoptosis. PI3K is a lipid kinase that activates AKT molecules. In turn, the activated AKT molecules stimulate mTOR, which promotes gene transcription and translation. This results in enhanced cell growth and proliferation, opposing cardiomyocyte hypertrophy (17, 18).

Previous studies did not reveal whether Apelin-13 plays an effect on cardiomyocyte hypertrophy through the PI3K-AKT-mTOR signaling pathway. Thus, it was imperative to carry out our study to elucidate the signaling mechanisms via which Apelin-13 protects the cardiovascular system against cardiomyocyte hypertrophy.

## Materials and Methods

***Antibodies, chemicals, and reagents***

Dulbecco’s Modified Eagle’s Medium (DMEM/High glucose) and Phosphate Buffer Saline (PBS) were purchased from Hyclone corporation, USA. Roche Corporation, USA, supplied the counting Kit-8 (CCK-8) solution. Phenylephrine (PE) and rapamycin (Rapa) were ordered from MedChemExpress cooperation, USA. Ten percent Tris Buffered saline Tween (TBST) and all primary and secondary antibodies were purchased from Abcam, UK. The Prime Script RT reagent kit and the SYBR Premix Ex Taq TM II kit were ordered from Takara, Japan. Ji Kai chemical technology cooperation, Shanghai, China, supplied plasmid GV493 and puromycin. The TRITC-conjugated phalloidin and 4’, 6-diamidino -2-phenylindole, blue (DAPI) were purchased from Suolaibao Technology Cooperation, Beijing, China. All other reagents and chemicals were ordered from Sigma-Aldrich, USA.

***Cell culture***

The H9C2 cell line, a subclone of the original clonal cell line derived from embryonic BD1X rat heart tissue, was obtained from Hunan Youcheng biological corporation. The cells were cultured in DMEM/High glucose (Hyclone corporation, USA) supplemented with 10% FBS, 100 U/ml penicillin, and 0.1 mg/ml streptomycin at 37 ^°^C in plates. Cell subculturing was performed when the cell confluency reached 80% to 90%. After a 48 hr incubation, cells were collected by centrifugation and stored at -80 ^°^C.

***Cell viability assay***

H9C2 cells were plated in a 96-well plate at a density of 2×10^4^ cells/well. Next, 10µl CCK-8 solution (Roche Corporation, USA) was added, and cells were cultured for 4 hr at 37 ^°^C in the dark. Finally, the absorbance was measured at 450 nm using a Universal Microplate Spectrophotometer (Bio-Rad Laboratories, Incorporated).

***Western blotting***

H9C2 cells were harvested for Western blotting at a density of 3-4×10^6 ^cells/well. Standard western blot assays were used to analyze the levels of the PI3K/AKT/mTOR pathway proteins and MYH7 in cells. The H9C2 cells were harvested and washed with PBS (Hyclone corporation, USA), and protein was extracted using cell lysis solution (Sigma, USA). A 12% separation adhesive and a 5% concentration adhesive were prepared, and the polyacrylamide gel was placed in an electrophoresis tank. Subsequently, samples were loaded, and gel electrophoresis was initiated. Next, the protein was transferred electrophoretically to polyvinylidene difluoride membranes (PVDF) (Sigma-Aldrich, USA). Hence, the membrane was blocked with 5% non-fat milk for 1hr before being incubated overnight at 4 ^°^C with primary antibodies against PI3K/AKT/mTOR and MYH7 protein (Abcam, UK). The following day, membranes were washed three times with 10% TBST (Abcam, UK) and subsequently incubated with secondary antibodies (Abcam, UK) labeled with horseradish peroxidase 8 and were developed via enhanced chemiluminescence. Protein levels were detected using a Bio-Rad image analyzer and densitometry analysis ware using Quantity One version 4.4 (Bio-Rad Laboratories, Inc, USA).

***Quantitative real-time PCR analysis***

RNA was extracted using an RNA isolation kit (Baiao innovation bioflux Cooperation, Beijing, China). RNA concentration and purity were assessed by the measurement of optical density at 260 and 280 nm. The purified MYH7 mRNA was reverse transcribed with Prime Script RT reagent kit (Takara, Japan), and quantitative real-time PCR was performed on Takara Real-time PCR instrument using the SYBR Premix Ex Taq TM II kit (Takara, Japan). The sequences of forward (F) and reverse (R) primers are listed in Table 1.

***Electroporation and plasmids transfection***

Plasmids (plasmid number: GV493, plasmid element sequence: hU6-MCS-CBh-gcGFP- IRES-puromycin, Ji Kai chemical technology cooperation, Shanghai, China) designed to interfere with the APJ expression were transfected to H9C2 cells by electroporation using electric transfection equipment (Celetrix Biotechnology cooperation, USA) according to the manufacturer’s protocols. Electroporation conditions were described as 640 v, 30 s. Then, 5 µg/ml puromycin (Ji Kai chemical technology cooperation, Shanghai, China) was supplemented to select the stable cell lines 48 hrs following electroporation transfection. After 10 days of cell culture, RT-PCR and Western blot were carried out to confirm and screen the APJ low expression in H9C2 cells (Si-APJ group) and the empty vector plasmid in H9C2 cells (Vector-Si-APJ control group).

***Fluorescence imaging***

TRITC-conjugated phalloidin (Suolaibao Technology Cooperation, Beijing, China) was used to stain and visualize filamentous actin and cytoskeleton. DAPI (blue, Suolaibao Technology Cooperation, Beijing, China) was utilized to stain the nucleus. Fluorescence imaging was performed with confocal microscopy (Leica, Germany), and analysis was performed with high content imaging software (Perkin Elmer, USA).

***Experimental grouping***

**Figure 1:**
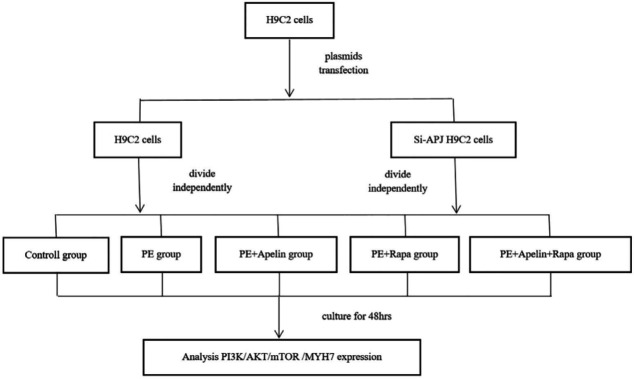
Experimental grouping and flow chart. Based on the mode of intervention, H9C2 cells and Si-APJ cells were divided independently into five groups as follows: the control group, the Phenylephrine (PE) group, the PE+Apelin group, the PE+Rapa group, and the PE+Apelin+Rapa group

The schematic diagram of experimental groupings is outlined in Figure 1. H9C2 cells and Si-APJ cells were divided independently into five groups as follows: the control group, the PE group, the PE+Apelin group, the PE+Rapa group, and the PE+Apelin+Rapa group considering the mode of intervention. Control group: H9C2 cells were cultured in a DMEM medium for 48 hr. PE group: H9C2 cells were cultured in a DMEM medium with phenylephrine (MedChemExpress cooperation, USA) for 48 hr. PE+Apelin group: H9C2 cells were cultured in a DMEM medium containing phenylephrine and Apelin-13 (Sigma, USA) for 48 hr. PE+Rapa group: H9C2 cells were cultured in a DMEM medium supplemented with phenylephrine and rapamycin (MedChemExpress cooperation, USA) for 48 hr. PE+Apelin+Rapa group: H9C2 cells were cultured in a DMEM medium with phenylephrine, Apelin-13, and rapamycin for 48 hr. 

***Statistical analysis***

All data were checked for normal distribution (Kolmogorov-Smirnov test) and homogeneity of variances (Levene test). All data on quantitative characteristics are presented as mean±standard error (X±SD). Independent-samples t-test was used for comparisons between the two groups. One-way analysis of variance (ANOVA) followed by Bonferroni’s *post-hoc* test was used to compare three (or more) groups. All analyses were performed using the software SPSS 22.0 (SPSS Inc., Chicago, USA). A two-tailed *P-value <*0.05 was considered statistically significant.

## Results

***The optimum concentration of PE-induced cardiomyocyte hypertrophy***

**Figure 2:**
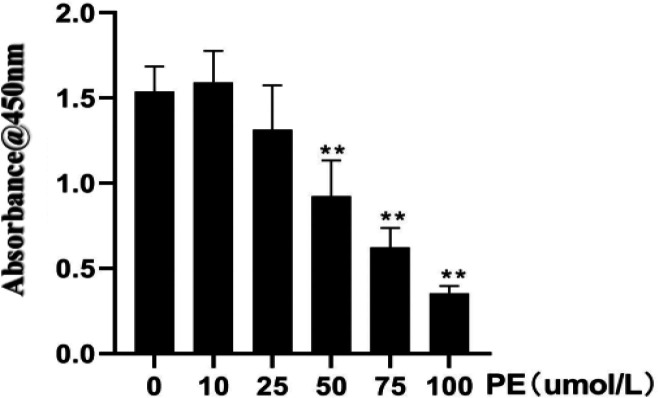
Different concentrations of phenylephrine and the viability of H9C2 cells (compared with the 0 µmol/l PE group, **P<0.01)

**Figure 3:**
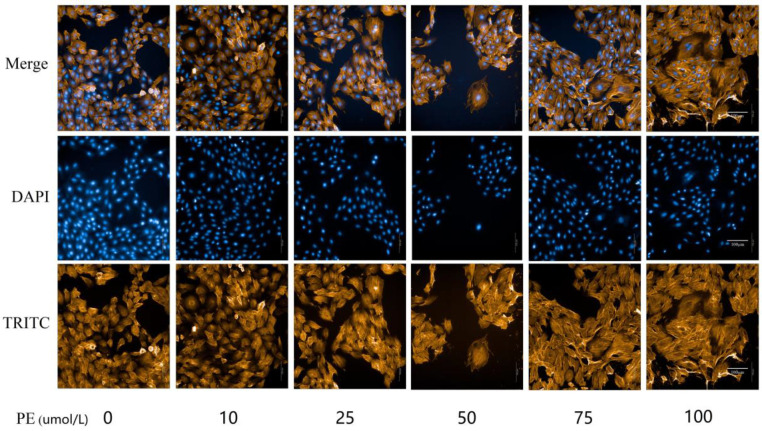
TRITC phalloidin cytoskeleton staining and DAPI nuclear staining under different concentrations of PE (magnification, ×400)

TRITC: TRITC phalloidin cytoskeleton staining; DAPI: DAPI nuclear staining; Merge: TRITC phalloidin cytoskeleton staining merged pictures with DAPI nuclear staining

**Figure 4:**
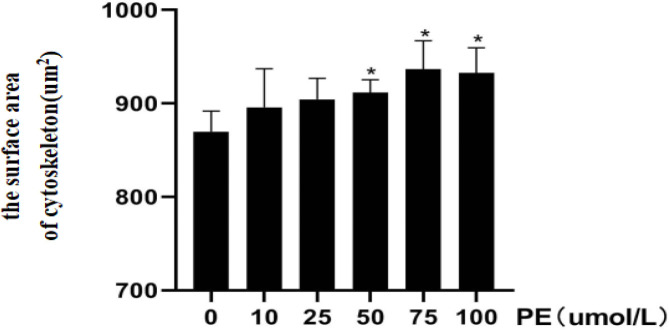
Different concentrations of phenylephrine and the surface area of the cytoskeleton(compared with the 0 µmol/l Phenylephrine group, *P<0.05)

**Figure 5:**
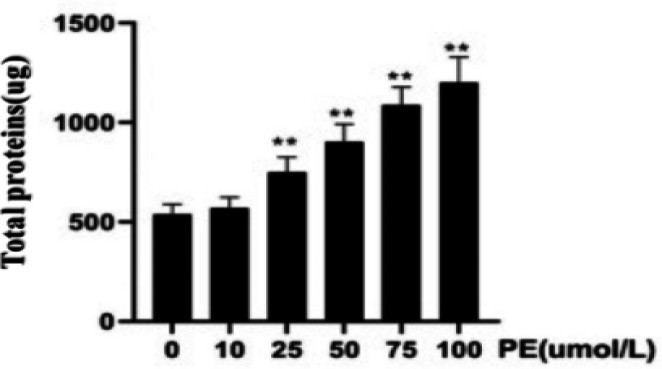
Different concentrations of phenylephrine and the total proteins of H9C2 cells (compared with the 0 µmol/l Phenylephrine group, **P<0.01)

**Figure 6:**
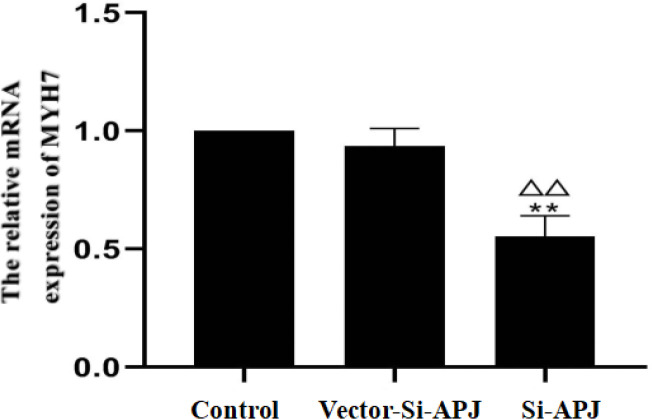
Different concentrations of phenylephrine and the relative mRNA expression of MYH7 (compared with the control group, **P<0.01; compared with the Vector-Si-APJ group, ^△△^*P<*0.01)

Different concentrations of PE (0, 10, 25, 50, 75, 100 µmol/l) were cultured with H9C2 cells, and the viability of H9C2 cells was examined via the CCK-8 absorbance assay. The cell viability results were as follows: 1.54±0.15, 1.59±0.19, 1.31±0.26, 0.92±0.21, 0.62±0.11, 0.35±0.04. Compared with the 0µmol/L PE group, the cell viability decreased gradually as the concentration of PE increased. The decline of cell viability in the 50 µmol/l group was relatively small and statistically significant, while the H9C2 cell viability in 75 µmol/l and 100 µmol/l groups was significantly inhibited (*P*<0.01, Figure 2). TRITC phalloidin staining and DAPI nuclear staining enable the visualization of the filamentous actin cytoskeleton and the nucleus of H9C2 cells, respectively. Figure 3 shows a direct relationship between the surface area of the cytoskeleton and the PE concentration. Compared with the 0 µmol/l PE group, the surface area of the cytoskeleton (911.58±11.20 µm^2^, 936.52±30.69 µm^2^, 932.56±23.51 µm^2^) increased gradually as the PE concentration increased (50 µmol/l, 75 µmol/l, 100 µmol/l, *P*<0.05, Figure 4). Compared with the 0 µmol/l PE group, the total proteins of H9C2 cells (750.87±74.87 µg, 904.12±87.33 µg, 1089.97±88.36 µg, 1202.31±126.99 µg) increased gradually as the PE concentration increased(25 µmol/l, 50 µmol/l, 75 µmol/l, 100 µmol/l, *P*<0.05, Figure 5). Regarding the MYH7 mRNA expression levels, compared with the 0 µmol/l PE group, the relative mRNA expression of MYH7 (1.84±0.35, 2.02±0.56, 2.03±0.52, 2.18±0.42) increased gradually as the PE concentration increased (25 µmol/l, 50 µmol/l, 75 µmol/l, 100 µmol/l, *P*<0.05, Figure 6). Altogether, these results provide a clear qualitative and quantitative understanding of cell viability, the surface area of the cytoskeleton, total protein, and the relative mRNA expression of MYH7 in H9C2 cells under different concentrations of PE. In addition, the optimal PE exposure concentration (*i.e., *50 µmol/l) was determined.

***The efficiency of plasmids electroporation and transfection***

**Figure 7:**
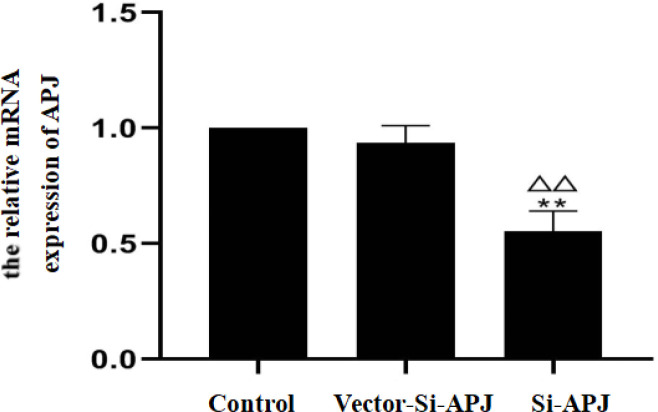
The relative mRNA expression of APJ in three groups (compared with the control group, **P<0.01; compared with the Vector-Si-APJ group, ^△△^*P<*0.01)

**Figure 8:**
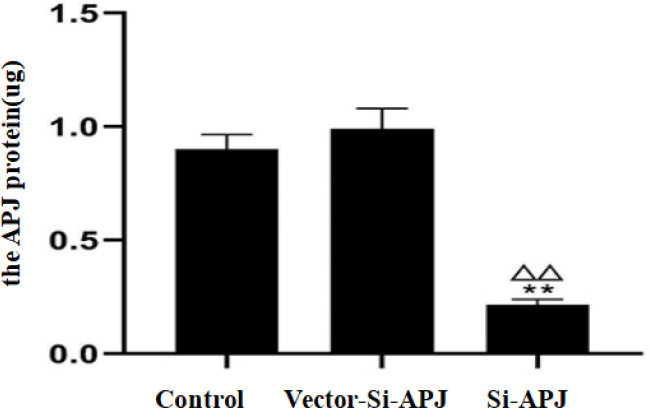
The APJ protein in three groups(compared with the control group, **P<0.01; compared with the Vector-Si-APJ group, ^△△^*P<*0.01)

The efficiency of plasmids transfection was detected after electroporation among the control, Si-APJ, and Vector-Si-APJ groups. The relative mRNA expression of APJ and the APJ protein in the Si-APJ group was 0.55±0.09 and 0.22±0.02 µg, respectively. Compared with the control group or Vector-Si-APJ group, the relative mRNA expression of APJ and the APJ protein in the Si-APJ group was significantly decreased (*P<*0.01). Conversely, compared with the control group, the decline of relative mRNA expression of APJ and the APJ protein in the Vector-Si-APJ group was not statistically significant (*P*>0.05, Figure 7 and Figure 8). 

***The optimum concentration of Apelin-13***

**Figure 9:**
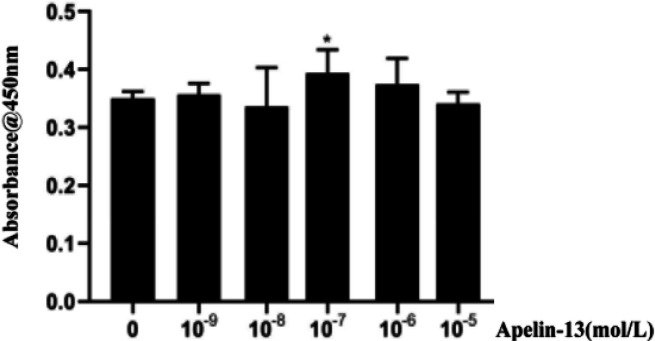
Different concentrations of Apelin-13 and the viability of H9C2 cells (compared with the 0 µmol/l Apelin-13 group, *P<0.05)

Different concentrations of Apelin-13 (0, 10^-9^, 10^-8^, 10^-7^, 10^-6^, 10^-5 ^mol/l) were cultured with H9C2 cells, and the viability of H9C2 cells was examined via the CCK-8 absorbance assay. Compared with the 0 µmol/l Apelin-13 group, the cell viability in the 10^-7^mol/l group was increased (*P*<0.05, Figure 9), so the optimal Apelin-13 exposure concentration (10^-7 ^mol/l) was determined.

***Effects of Apelin-13 on the PI3K/AKT/mTOR*** ***signaling pathway***

**Figure 10:**
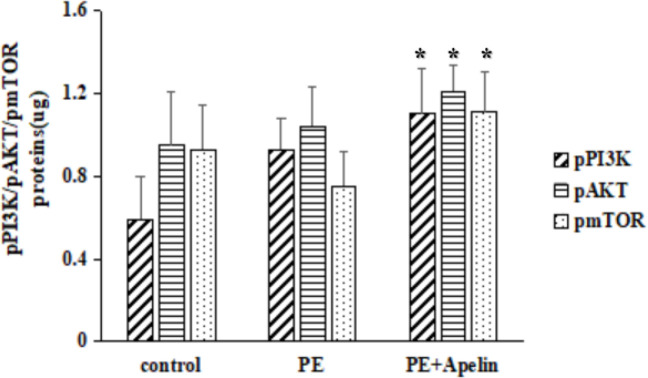
Phosphorylation PI3K/AKT/mTOR proteins in H9C2 cells (compared with the Phenylephrine group, *P<0.05)

**Figure 11:**
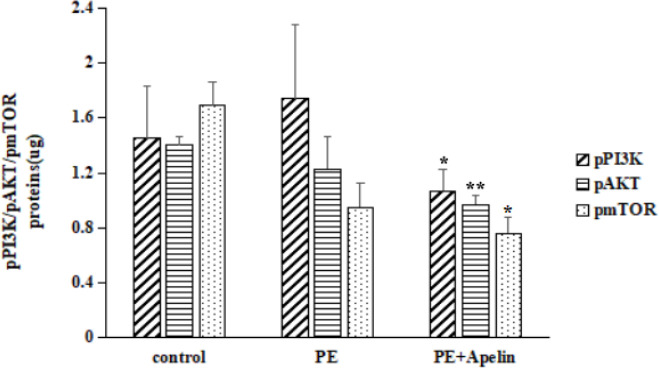
Phosphorylation of the PI3K/AKT/mTOR proteins in Si-APJ H9C2 cells (compared with the Phenylephrine group, *P<0.05, **P<0.01)

In H9C2 cells, there was no significant difference in the phosphorylation of the PI3K/AKT/mTOR pathway proteins between the control group and the PE group. Compared with the PE group, the phosphorylation of the PI3K/AKT/mTOR pathway proteins in the PE+Apelin group was significantly increased (Figure 10, *P*<0.05). In contrast, in Si-APJ H9C2 cells, there was no significant difference in the phosphorylation of the control group and the PE group in the PI3K/AKT/mTOR pathway proteins. However, compared with the PE group, phosphorylation of the PI3K/AKT/ mTOR pathway proteins in the PE+Apelin group was significantly decreased (Figure 11, *P*<0.05).

***Effects of Apelin-13 on MYH7 protein***

**Figure 12:**
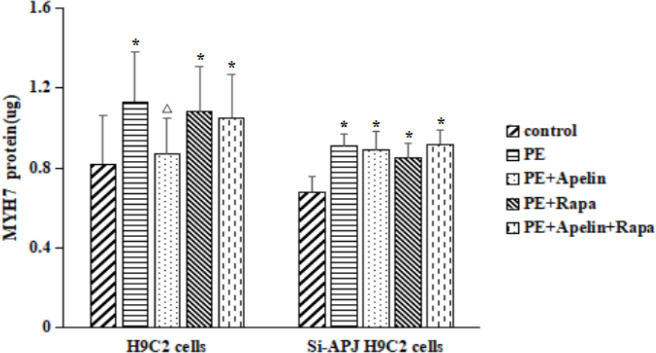
Effects of Apelin-13 on MYH7 protein in H9C2 cells and Si-APJ H9C2 cells (compared with the control group, *P<0.05; compared with the Phenylephrine group,^△^*P<*0.05)

Compared with the control group (1.01±0.24 µg), the MYH7 protein levels in the PE group (1.13±0.25 µg), PE+Rapa group (1.08±0.23 µg), and PE+Apelin+Rapa group (1.05±0.22 µg) was significantly increased (Figure 12, *P*<0.05) in H9C2 cells. In the same cell line, there was no significant difference in the MYH7 protein levels between the control group and the PE+Apelin group (0.87±0.18 µg). Compared with the PE group, the MYH7 protein in the PE+Apelin group was significantly decreased (Figure 12, *P*<0.05). In contrast, there was no significant difference in the MYH7 protein levels between the PE+Rapa and the PE+Apelin+Rapa groups. 

In Si-APJ H9C2 cells, compared with the control group (0.68±0.08 µg), the MYH7 protein levels were increased in the following groups (Figure 12, *P*<0.05): PE (0.91±0.06 µg), PE+Apelin (0.89±0.09 µg), PE+Rapa (0.85±0.08 µg) and PE+Apelin+Rapa (0.92±0.07 µg). In the same cell line, there was no significant difference in the MYH7 protein levels among the other four groups.

**Table 1:** Quantitative RT-PCR primer sequences of MYH7 and β-ACTIVE

Primer name	5’-3’sequences
MYH7-F	CAGAACACCAGCCTCATCAAC
MYH7-R	TCTCCTCTGCGTTCCTACAC
β-ACTIVE-F	CGCGAGTACAACCTTCTTGC
β-ACTIVE-R	CCTTCTGACCCATACCCACC

APJ: Angiotensin type 1 receptor

APJ: Angiotensin type 1 receptor

APJ: Angiotensin type 1 receptor

APJ: Angiotensin type 1 receptor

APJ: Angiotensin type 1 receptor

APJ: Angiotensin type 1 receptor

## Discussion

Apelin, originally identified in the bovine stomach, is the endogenous ligand of APJ. Together, they form the Apelin/APJ system, which is distributed in cardiovascular, central nervous, respiratory, and other systems and seems to be involved in the regulation of food intake, energy metabolism, cardiovascular system, angiogenesis, and neuroendocrine functions and more (19, 20). The Apelin gene is located on chromosome Xq25-26.1 and composed of 2 introns and 3 exons encoding a 77 amino acids-long protein. Apelin/APJ is expressed in endothelium and smooth muscle cells and is present in secretory organelles, such as the Golgi apparatus, the endoplasmic reticulum, and secretory vesicles. The gene encoding the apelin shares the 31% homology with the angiotensin II type 1 receptor, and the apelin peptide competitively bound to the angiotensin II type 1 receptor, exerting vasodilator effects (21). The apelin propeptide is abundant in basic amino acid residues, which are readily endopeptidase cleavage sites and gives rise to several bioactive C-terminal fragments with different biological functions, such as Apelin-10, Apelin-11, Apelin-12, Apelin-13, Apelin-15, Apelin-17, Apelin-36. Among these, Apelin-13 has the most potent beneficial effects on the cardiovascular system (antihypertrophic and vasodilating effects), drawing widespread attention from cardiovascular researchers. It has been reported that Apelin-13 could reduce cardiac hypertrophy, alleviate myocardial apoptosis and optimize hemodynamics (22). 

Cardiac hypertrophy is characterized by myocardial cell hypertrophy, extracellular matrix increase, and myocardial interstitial cell proliferation. It is a compensatory response to a variety of cardiovascular diseases, such as hypertension and heart valve disease, and is a powerful predictor of adverse cardiovascular prognosis (23). To date, two methods have been mainly used to produce cardiac hypertrophy models: I) To establish a cardiac hypertrophy model by adrenaline or surgery in an animal model. II) To induce cardiac hypertrophy at the cellular level by angiotensin II, isoproterenol, or PE (24). It is considered that apelin is the ligand of the angiotensin type 1 receptor relative receptor protein, which might interact with angiotensin II. PE is a selective α1-adrenoceptor agonist, which causes cardiac hypertrophy by increasing vascular resistance and reducing vascular compliance. Previously, PE had been successfully used to construct cardiomyocyte hypertrophy models *in vitro *(25, 26). Therefore, the PE-induced cardiac hypertrophy model *in vitro* was established in H9C2 cells in our study. The results of this study showed that after culturing H9C2 cells with different concentrations of PE, the surface area of H9C2 cells, the total protein, and the relative expression of MYH7 mRNA all increased along with the increase in PE concentration. This suggested that the PE-induced cardiac hypertrophy model *in vitro* was successful and that the 50 µmol/l condition was the best working concentration of PE. A concentration gradient assay was performed to determine the optimal concentration of Apelin-13 in this study. The result showed that the cell viability in the 10^-7 ^mol/l group increased in agreement with previous studies (27), and the optimal Apelin-13 exposure concentration was determined to be 10^-7 ^mol/l. 

Previous studies by Chen L. had shown that pinoresinol diglucoside could inhibit isoproterenol-induced cardiac hypertrophy through AKT/mTOR signaling pathway (28). It has been confirmed that the Krüppel-like factor could inhibit isoproterenol-induced cardiac hypertrophy through AKT/mTOR signaling pathway (29). Also, it has been clarified that the overexpression of 5-hydroxytryptamine receptor 2A was involved in cardiac hypertrophy through the AKT/mTOR signaling pathway (30). Therefore, many studies have confirmed that activating AKT/mTOR signaling pathways could potentially reduce cardiac hypertrophy. This study detected a reduced level of phosphorylated PI3K/AKT/mTOR pathway proteins in cardiac hypertrophic cells. In contrast, Apelin-13 combined with APJ could increase the expression of PI3K/AKT/mTOR phosphorylated proteins in cardiac hypertrophic cells. The study of Si-APJ H9C2 cells, deficient in the expression of APJ, found that Apelin-13 did not increase the phosphorylation levels of PI3K/AKT/mTOR. This result further supported the finding that Apelin-13 could exert its biological effect of inhibiting cardiac hypertrophy by increasing the phosphorylation of the PI3K/AKT/mTOR signaling pathway. This study also observed that following rapamycin addition to inhibiting the activity of mTOR activity, the effect of Apelin-13 on improving cardiac hypertrophy was significantly reduced and that the expression of MYH7 was significantly increased. This also suggested that Apelin-13 could improve cardiac hypertrophy by activating PI3K/AKT/mTOR signal pathway. 

The results from this study bridged a gap in the knowledge of the mechanism of Apelin-13 improving cardiac hypertrophy. However, this study also carried some limitations. Firstly, H9C2 cells were not primary cultured cells, which could only partly simulate some characteristics of cardiomyocytes. Secondly, we could not distinguish atrial or ventricular cells, and there was no further inhibition of PI3K and AKT. In the future, these aspects need to be addressed by performing primary cell culture, utilizing additional signalling pathway inhibitors, and conducting animal experiments to confirm the findings.

## Conclusion

Apelin-13 can reduce PE-induced cardiomyocyte hypertrophy by activating the PI3K/AKT/mTOR signaling pathway. Activating Apelin/APJ system is expected to be a target for treating cardiomyocyte hypertrophy.

## Authors’ Contributions

HF, ZPL, and RJM Designed the experiments; YP and CSW Performed experiments and collected data; YP, RJM, and ZPL Discussed the results and strategy; HF, RJM, and ZPL Supervised, directed, and managed the study; YP Wrote the article; RJM Performed critical revision or editing of the article; YP, RJM, CSW, HF, and ZPL Approved the final version to be published.

## Conflicts of Interest

The authors declare no financial or commercial conflict of interest.
